# Global hepatitis B and D community advisory board: expectations, challenges, and lessons learned

**DOI:** 10.3389/fpubh.2024.1437502

**Published:** 2024-08-26

**Authors:** Fiona Borondy-Jenkins, Bright Ansah, Jacki Chen, Amanda Goldring, Yasmin Ibrahim, Shaibu Issa, Silvana Lesidrenska, Tanya Machado, Holly Moore, Richard Njouom, Prince Okinedo, Rhea Racho, Lori Scott, Beatrice Zovich, Chari Cohen

**Affiliations:** Hepatitis B Foundation, Doylestown, PA, United States

**Keywords:** community empowerment, research participation, global advisory board, clinical trial design, drug development, hepatitis B, hepatitis D, patient centricity

## Abstract

**Introduction:**

Community Advisory Boards (CABs) play an important role in developing and delivering patient-centered care. However, the impact of participation on CAB members has not been well studied, particularly on the global scale. In 2022, the Hepatitis B Foundation (HBF) convened the first global hepatitis B and hepatitis delta CAB with 23 members from 17 countries, representing six out of the seven World Health Organization (WHO) regions, and countries with the largest hepatitis B and hepatitis delta disease burden.

**Methods:**

To reflect on the process of assembling an effective and motivated CAB and assess the impact on CAB participants, three virtual focus group sessions were held with 16 participants in July and August 2023. Sessions were recorded and transcribed. Questions focused on motivations for joining the CAB, membership experiences, and lessons learned. Grounded theory analysis was used to generate hypotheses about reasons for CAB members’ participation, as well as challenges and suggestions. Qualitative analysis using inductive reasoning identified key themes within responses. Transcripts were independently analyzed by a primary and secondary coder.

**Results:**

Motivations for joining the CAB included participants’ desire to advocate for people living with hepatitis B and hepatitis delta, and other altruistic factors. Participants reflected that through CAB membership, they gained networking and advocacy opportunities and enhanced their hepatitis B- and hepatitis delta-related knowledge. Challenges participants experienced were related to time, physical limitations, and stigma. Finally, participants discussed their limited direct engagement with drug developers and proposed ways the CAB can increase interactions with stakeholders going forward.

**Discussion:**

Based on participants’ assessments, establishing a global CAB for stigmatized infectious diseases is worth the effort. Regular internal review of community advisory boards’ structure and performance is critical to ensure the CAB is fulfilling its mission.

## 1 Introduction

According to the World Health Organization (WHO), there are 254 million people living with chronic hepatitis B virus (HBV) (1), and an estimated 12.7 million people living with hepatitis delta (HDV) worldwide (2). Hepatitis B is the most common serious liver infection in the world, but is preventable with a vaccine, and treatable with drug therapies. HBV heavily affects those in high endemic areas including in some parts of Asia, the Western Pacific, Africa and Eastern Mediterranean countries (1, 3). Hepatitis delta affects between 5 and 10% of individuals living with HBV. Because HDV depends on HBV to survive and reproduce, it is also preventable with the hepatitis B vaccine. Although HDV is less common, it is the most severe form of viral hepatitis and is seen most frequently in Mongolia, Moldova, Romania, and countries in Western and Central Africa (4). In the United States, both HBV and HDV are more frequently seen in populations that were born in regions of high endemicity (5).

Historically, patient preferences and insight have not been rigorously included in the development of HBV/HDV clinical trials. However, with many new HBV/HDV clinical trials being initiated, now is the ideal time to engage with people impacted by HBV/HDV. In keeping with researchers’ efforts to design precise and holistic clinical trials, patient experiences must be incorporated to ensure outcomes are appropriate and effective (6). This can only be achieved by engaging people with lived experience of the disease in the entire process, from conceptualization and design to follow-up. Collaborative efforts between researchers and affected populations are necessary to identify and address key clinical needs that are most relevant to patients.

Community advisory boards (CABs) provide a well-established structure for community engagement through fostering collaborative partnerships with researchers to integrate patient perspectives on pertinent issues (7). Patient insights are crucial for enhancing the design and acceptance of clinical trials (8) and ensuring potential participants’ comprehension of research practices, such as the informed consent process (7). CABs offer community members a platform to express concerns and recommend research approaches that are respectful to their community (9). CABs tailored for diseases such as HIV/AIDS (10, 11) and tuberculosis (12) have demonstrated success in promoting participatory research and designing person-centered clinical trials that align with the preferences of affected communities. Limited documented strides have been made in the areas of HBV and HDV.

In 2022, the Hepatitis B Foundation (HBF) established a global CAB, composed of people living with hepatitis B and delta (PLHB and D), as well as caregivers and advocates, to increase engagement between these communities and those working in the drug development and clinical trial spaces, and to amplify the voices of those most affected by these viruses. Upon conception of the CAB, the research team sent out an open call for applications that was widely distributed through social media channels, email listservs and website postings, as well as through an established network of patients, providers and community advocates in the HBV/HDV space. Those interested were invited to apply to join the CAB. Founding members were selected based on certain qualifications, including but not limited to their HBV/HDV status or being a caregiver to someone living with HBV/HDV, English proficiency and their experience in HBV/HDV education and advocacy efforts. Joining the CAB is voluntary, and those who chose not to submit an application were not considered to join. As the founding cohort of CAB members are nearing the end of their first two-year term, HBF sought to document the process of assembling a highly involved CAB, including members’ motivations for joining, their membership experiences, and any lessons learned regarding their interactions with drug developers and other stakeholders.

In contrast to most existing literature about the role of CABs, which focuses on the healthcare space, this qualitative study presents the benefits and challenges of a global CAB within the public health and clinical research space, as well as potential suggestions for deepening engagement. Documenting the inaugural cohort’s experiences and the initial challenges, can provide guidance the current CAB’s sustainability efforts and to others seeking to embark on similar endeavors to build robust cohorts of global advocates who can transform the drug development process into one that is truly patient-centered.

## 2 Methods and materials

In July and August 2023, three virtual, 90-min focus group sessions were facilitated by trained moderators in English, using Zoom. All CAB members were invited to participate. Of those who participated in the focus groups, there were 10 HBV and six HDV CAB members. Two experienced, external third-party moderators facilitated the three focus groups to reduce the risk of social desirability bias. One researcher joined each focus group as a silent observer, to provide technical support and take notes during each session. With participant consent, sessions were audio-recorded and professionally transcribed.

Grounded theory analysis was used to generate hypotheses about participants’ anticipated responses. This is a qualitative research method in which empirical data is collected first, and then researchers create a theory ‘grounded’ in the results of the data. This is often used to analyze social processes (13). This method informed the creation of a focus group discussion guide (Supplementary Figure 1) (13). There were five sections in this guide, spanning their understanding about the CAB’s goals, their motivations for joining the CAB, their experiences, their thoughts about drug development and any lessons learned.

Qualitative analysis using inductive reasoning was used to identify key themes within responses, which along with the guide, informed the creation of a codebook (Supplementary Table 2). Each transcript was independently coded by a primary and secondary coder using the qualitative analysis software NVivo Version 13 (14). Intercoder agreement, or reliability, is measured by computing for the Kappa coefficient, a statistical test that offers the measure of agreement between different coders’ categorization of data and controls for the random agreement factor (15, 16). The final kappa coefficient was 0.80, indicating high inter-coder reliability.

### 2.1 Ethical considerations

Prior to their participation, participants were made aware of the possibility that their identity might not remain confidential, as their membership in the CAB is public information and thus disclosure of HBV/HDV status was a potential risk of focus group participation. This study was approved by Heartland Institutional Review Board (HIRB) (HIRB Project No. 081122-407).

## 3 Results

### 3.1 Participant characteristics

Across the three focus groups, there were 16 participants who represented 11 different countries. Table 1 presents the demographic characteristics of participants.

**Table 1:** Participant demographics, 2023.

Demographics	Totals *N* (%)
Age range	
25–35	4 (25%)
36–45	5 (31.25%)
46–55	3 (18.75%)
56–65	2 (12.5%)
65 and above	2 (12.5%)
Gender	
Male	11 (68.75%)
Female	5 (31.25%)
Ethnicity	
Asian	4 (25%)
African	7 (43.75%)
Caucasian	3 (18.75%)
Eastern European	2 (12.5%)
Hepatitis diagnosis	
Hepatitis B	10 (62.5%)
Hepatitis delta	6 (37.5%)
Residing WHO region (country)	
African region	5 (31.25%)
Cameroon	1
Ghana	1
Nigeria	1
Tanzania	1
Uganda	1
Western Pacific region	2 (18.75%)
Australia	1
Philippines	1
European region	3 (18.75%)
Bulgaria	1
Romania	1
United Kingdom	1
Region of the Americas	6 (37.50%)
USA	6

Table represents the demographic makeup of study participants, including ethnicity and country of residence.

### 3.2 Findings

Additional quotes for each theme can be found in Supplementary Table 1.

#### 3.2.1 Motivations for joining the CAB

Participants expressed many motivations for joining the CAB; representing PLHB and D, altruism [participants’ motivation to do something to benefit others, rather than, only themselves (17)], and educating their community were most frequently cited as the primary motivators.

##### 3.2.1.1 Representing PLHB and D

Most participants agreed that they wanted to be the voice of PLHB and D, to bring patients’ perspectives to the forefront in drug development, policy, and advocacy efforts. Many also expected to raise awareness of the challenges these groups face worldwide. In the words of one participant from the U.K.:

*I think being a member of [the] CAB gives us that voice to speak out, not just for ourselves, but to speak out for so many people who have no voice at all… being their voice to the pharmaceutical companies and shouting loud and clear not just for ourselves but for everybody with hepatitis B [and D].*

##### 3.2.1.2 Altruism

Altruism was another motivating factor for CAB membership. Many participants explained how they would be pleased if their advocacy efforts in the drug development process would lead to a cure for HBV and effective treatments for HDV for future generations, even if they did not personally benefit. A Romanian participant summarized this:

*Of course, you hope to do it for yourself, for your friends, for your family. But then also for the next generations because, yeah, I wouldn't mind, if I would go away and just disappear, but then maybe the next generation or the two generations, hepatitis B and D would just be like some flus and that would be good … even if it didn't help my generation.*

Participants hoped that they would be able to improve peoples’ lives through community education, to reduce stigma associated with hepatitis B and D, and to increase screening and vaccination rates. This idea was summarized by a U.S. participant (#1), *“I want to educate my family, my friends, the community about viral hepatitis, which you know, if people are educated, there’d be a lot less of it because at least for hepatitis B, there are vaccinations.”*

##### 3.2.1.3 Knowledge and skill-building

Participants also joined the CAB because they wanted to build their knowledge and improve their advocacy skills to make a difference in a global context. Specifically, participants emphasized their desire to advocate for PLHB and D by bringing more medical and research resources to the African context. One participant from Cameroon stated, *“Do not forget Africa”* when working toward hepatitis elimination efforts. A participant from Tanzania also shared, *“Who can break the silence is us. We need to tell our government that there is a problem, and we need to end this problem. And the best people with knowledge … can come from this CAB.”*

#### 3.2.2 Membership reflections

When reflecting on their membership in the CAB, participants cited positive outcomes, including building greater confidence and developing global connections, however, some have cited personal challenges.

##### 3.2.2.1 Experience as a CAB member

Many participants highlighted increased confidence when advocating for PLHB and D, as expressed by a participant from the U.K., *“I was diagnosed with HBV three and half years ago and for a long time I felt I had no voice whatsoever. And I think being a member of [the] CAB gives us that voice to speak out.”* A participant from Nigeria added:

*Frankly, this [CAB] has really impacted positively in the way I carry on with hepatitis advocacy. I used to be this very shy person when it comes to reaching out to people. But somehow this has emboldened me and given me a lot of confidence.*

Other positive experiences included feeling empowered to raise questions and propose solutions during internal CAB discussions. For example, a participant from the Philippines said, *“The discussions are really more important to me because it is where we can raise our questions and give our suggestions and opinions.”*

Many also felt that their affiliation with a global advisory board lent greater credibility to their local advocacy endeavors. One Nigerian participant articulated, *“When I do my advocacy work and I talk to people in government and I let them know that I’m a member of CAB, they really want to listen. And I think that’s a huge support for me.”*

Conversely, others felt the CAB is not acknowledged by pharmaceutical companies, policymakers, and government agencies even with the Foundation’s support. Many felt this inhibits their ability to provide important insights about PLHB and D’s preferences in drug development. A U.S. participant (#2) remarked:

*I think the barrier is enormous. [It is not easy for us] to interact with the influential stakeholder[s] like the government agency, the policymaker. We rely on the Hep B Foundation to be able to give us a sort of a seat there that we can communicate with them. I'm hoping that some of the stakeholders will be able to pay more attention to individual patients' voices, or as a CAB member that we have more chance[s] to interact with them.*

##### 3.2.2.2 Global team membership

Participants discussed how the CAB has equipped them with newfound global collaboration opportunities, and how this has benefitted their personal advocacy endeavors. A Nigerian participant underscored the breadth of the international relationships fostered through the CAB: *“I’ll be organizing a summit in my State, and some of the CAB members will be featured as guest speakers.”*

Additionally, a participant from Ghana explained how the cooperative nature of the CAB has provided them with global learning opportunities: *“I’m feeling comfortable honestly because [I am] learning from people [who are] globally bringing their experiences [and] sharing what they think.”*

##### 3.2.2.3 Building knowledge

Participants appreciated the knowledge gained through training, discussions, and presentations from guest speakers. A U.S. participant (#3) explained how the required trainings enhanced their understanding of drug development processes:

*To me the most important aspect was having training[s] on the drug discovery process. Many of us patients, we are always concerned about when [a] treatment or cure will be available. But what we tend to forget is finding treatment[s] or finding a cure is not a straightforward line.*

##### 3.2.2.4 Personal challenges

Some participants cited difficulties that accompany the coordination of a global group of individuals with lived experience, including identifying meeting times that are convenient for all time zones and making travel accommodations for PLHB and D, especially those who are limited due to physical ailments. One U.S. participant (#3) explained, *“I battle severe chronic fatigue, so it’s hard sometimes to travel.”* Additionally, there is the added level of fear about stigma and discrimination that comes with disclosing one’s disease experience, as expressed by a U.S. participant (#4):

*Why is it so difficult to be an advocate for this disease? It's because a big part of [it is] the unseen, the stigma, prejudice and discrimination. So, for me it's trying to find that yes, I want to contribute to this cause and let me share as much as I can to support that. At the same time, if this information gets shared somewhere, sometimes people are not understanding and there are consequences or risks that I end up taking.*

#### 3.2.3 Challenges and suggestions for increasing CAB engagement with industry

Participants reflected on the barriers they faced when trying to establish relationships with industry stakeholders. Participants suggested ways the CAB could increase interaction with drug and clinical trial developers and highlighted the body’s potential to contribute to the development of patient-centric clinical trials and effective drug outcomes.

##### 3.2.3.1 Engagement with drug and clinical trial developers

Engagement with drug developers proved to be one major and unanticipated difficulty in the inaugural year of the CAB. Many participants felt that drug developers did not take them seriously, as one participant from Romania stated, *“But then when we go and interact with the [drug developers], we have to somehow be [an equal player] for them also to be taken seriously.”* Another participant from Bulgaria was also eager to establish relationships with developers, *“So, if there is a possibility or some hidden keys, how to communicate with stakeholders openly and without any restriction, I’m open to hear.”*

Many participants proposed ideas for cultivating stronger relationships with researchers and drug developers going forward. One recurring thought was to build a larger presence, as representatives of PLHB and D, at the conferences and events attended by drug and clinical trial developers. A Romanian participant proposed:

*For us, what to do is just to be a powerful organization with a lot of members and be vocal and present at whatever world events we might hear about, it doesn't matter if it's [a conference] in Tanzania or in Paris, just be there with a member to have our voices and our organized ideas heard.*

##### 3.2.3.2 Unique contributions of CAB members

Participants presented various suggestions about how their insights could be useful to those working in the drug development and clinical trial spaces. Chief among these was the simplification of language to ensure that study materials are not burdensome for clinical trial participants to understand. One U.S. participant (#3) noted, *“…the way they are written out sounds like it’s written just for the pharmaceutical company and not for the patient. Because half of the thing you read is like you cannot make any sense of it.”*

Additionally, participants referenced their ability to serve as ambassadors to the larger patient community, bridging the gap between scientists and PLHB and D. In particular, many noted their potential to enhance recruitment efforts by assuaging the community’s apprehension of research. A Bulgarian participant remarked:

*Many patients are afraid to participate in clinical trials… So, I think one thing we can do [is] develop materials that are in [an] understandable language just to spread the word…patients don't realize that to have [an] approved drug, a clinical trial should be done with people. I mean, they are all afraid, but they are awaiting the drug, but we need to participate in clinical trials, otherwise we don't have the drug.*

Participants felt that their dignity and personhood could be lost when taking part in a study. To overcome this, one participant from the U.K. explained how their interactions with clinical trial developers could serve as a reminder of study participants’ humanity, *“I’m sure that when they are developing clinical trials, they do not necessarily see a human being at the end, if that makes sense? And [we can] represent, you know, it’s a human being, it’s not just a patient.”*

Participants also felt that as PLHB or D and CAB members, they are in a unique position to help drug and clinical trial developers understand patient perspectives globally and create more patient-focused clinical trials. For example, when reflecting on the consequences of siloed drug development processes, one participant from Ghana posited, *“It could be one [drug] that is going to be effective in Europe [but] when you bring it in Africa it’s going to work otherwise. So, I think they should also consider if that one is going to work.”* Another example, discussed by a U.S. participant (#3), was helping drug developers have greater involvement in addressing the multi-faceted barriers to participation to enhance clinical trial accessibility:

*Sometimes patients have concern with side effects, sometimes they have problems with transportation…So just having a conversation with the pharmaceutical company about all these challenges and finding a better solution that can fit in very well, I think will go a long way by having a lot of patients participate in the clinical trials.*

## 4 Discussion

Participants have shown enthusiasm for and dedication to being a part of the hepatitis B and delta CAB and have expressed that CAB participation has positively impacted their lives. This invokes a charge to continue working with CAB members to advance patient-centered hepatitis B and delta clinical trials and to enhance member capacity, engagement with stakeholders, and advocacy opportunities across the hepatitis B and D space.

### 4.1 Expectations

Collectively, CAB members shared that most expectations they had prior to joining had materialized, especially those related to internal and external opportunities for global collaboration. Many participants expected that they would be able to improve peoples’ lives through community education and reduce the stigma associated with hepatitis B and D, an experience which is personal for many members. To this end, the CAB has already undertaken projects and activities to increase community awareness through education and encouraging screening, vaccination, and treatment, strategies that have been found to effectively reduce HBV transmission and mortality rates (18).

CAB members were motivated to join because of their desire to advocate for PLHB and D and to amplify patient voices within the drug development and policy spheres. Additionally, participants were interested in raising global awareness regarding the country-specific challenges faced by PLHB and D. CAB members are well equipped to do this, as they have strong support from and are trusted by their communities and have been recognized at regional and national levels for their efforts. Success in amplifying community members’ voices has been documented previously, such as when an American Indian CAB raised researchers’ awareness about the community’s environmental challenges to healthy lifestyles prior to an intervention’s implementation (19).

The CAB members, especially from countries with limited resources, are aware of the power inequity. Instead of focusing on the disparity, they have chosen to use the CAB as a platform, to leverage their association with a western organization. This alliance allows for greater support, better resources, adding value to their advocacy efforts and what they can do for their communities.

Participants emphasized their desire to contribute to finding a cure for HBV and effective treatments for HDV as motivation for joining the CAB. Many believed their advocacy efforts would help lead to a cure and treatments for future generations and were passionate in their efforts, even if these outcomes did not materialize during their lifetimes. Altruism has previously been identified as an influential factor for research participation (20, 21), and within other advocacy groups championing hepatitis C, tuberculosis and HIV treatments (22). CAB participants’ dedication to helping PLHB and D illustrates how, despite being a diverse and global body, those with lived experience of a disease often share common goals which allows for membership cohesion.

### 4.2 Experiences

Focus group findings indicate a strong desire for those with lived experience to continue to learn, advocate, and connect through groups such as CABs. The global nature of the CAB contributed to CAB members’ experiences. By leveraging the CAB as a global forum, participants can collaborate on initiatives spanning multiple countries, and nurture global networks in their educational and advocacy pursuits concerning hepatitis B and D. This collaborative atmosphere fosters a sense of community and solidarity among members, enabling them to learn from each other’s diverse country-specific experiences, challenges and perspectives while navigating cultural nuances in communication and advocacy efforts across different countries. The importance of regional and global networks and replicating evidence-based models for patient advocacy has been demonstrated in other disease areas including breast cancer (23) and Pompe disease (24).

Given the goal of ultimately enhancing interactions between drug developers and patients represented by the CAB, one common theme was the value of learning the process behind treatment development and testing. Participants explained how they want to continue learning about the HBV/HDV research space, not only to improve their own advocacy skills, but also to educate their communities about the diseases. As identified in this study and previously, the capacity for individual advocacy efforts depends on accurate knowledge (25). Before joining this CAB, not all members had access to reliable information nor the chance to discuss HBV/HDV-related queries with trained healthcare professionals. One of the strengths of this CAB lies in its dismantling of this inequity by providing all members with the same learning opportunities, which has resulted in greater confidence among members when advocating for PLHB and D concerns across local and international platforms. This and previous research have illustrated the numerous ways in which patients can be involved in all aspects of the research process, and with training, can provide great support to any research initiative (26). Given participants’ dedication to ongoing learning and advocacy skill-building, they are well-positioned to enhance community understanding of HBV and HDV and acceptance of possible new treatments, and possibly garner future political support for new patient-centric guidelines for screening, treatment, drug development, and care as has happened previously in the case of HIV/AIDS (27). Stronger political support from various stakeholders and increased funding availability could further encourage the insights and experiences of PLHB and D to be incorporated into further research, drug development, and access to care across the globe. Furthermore, most CAB members who participated in this study lead local and national organizations that are well recognized in their respective countries. Their advocacy and expertise will certainly contribute toward the sustainability of this CAB.

A disappointing reality of the CAB’s journey so far has been difficulty engaging directly with drug developers. This has been interpreted by many members as a reluctance on the part of drug developers to consider the perspectives of those with lived experience in the research, discovery, and trial process. This experience differs from those described in existing literature, but this seems to be because much of the previous work done with CABs has included the establishment of a CAB in direct association with an existing research institute, rather than with the support of an independent disease advocacy organization, as is the case in the present study. Previously, many CABs have been established only for a particular project (28), a largely transactional experience which fails to nurture relationships between members and researchers and to center the patient voice in the drug development process. This underscores the benefits of a long-term, self-sustaining advocate group. Part of the future CAB journey will include establishing the role of this independent CAB among developers in the drug development and clinical trial spaces. Results of this assessment suggest that CAB members are already identifying possible strategies for improved future engagement, including helping researchers understand the unique benefits of engaging with an informed group of people with lived experience.

### 4.3 Solutions and suggestions

Participants identified strategies to enhance their interaction with each other as a team, and with the drug development industry. To overcome the challenge of coordinating meetings that accommodate those in many different time zones, participants have highlighted the benefits of alternating meeting times each month. This has been adopted within the CAB and most members have noted an increase in effectiveness and engagement.

When reflecting upon factors for which clinical trial developers should account when developing person-centered and inclusive clinical trials, participants were most concerned with expanded access, accurately assessing drug effectiveness in different settings, and quality-of-life considerations of potential treatments. To improve industry engagement in pursuit of these goals, participants suggested continued presence in medical and pharmaceutical spaces, emphasizing the importance of attending related events and conferences to represent PLHB and D. It was suggested that CAB members could even be involved in hosting such events to provide ongoing insight and consideration into the needs and preferences of PLHB and D. Patient involvement in the planning of industry conferences has been intermittent since 1992 and has led to improved patient engagement in all aspects of healthcare and inclusion of patient-centered issues in research agendas (29).

The FDA has recently issued a “Diversity Action Plan to Improve Enrollment of Participants from Underrepresented Populations in Clinical Studies” (30). This is to enhance clinical trial diversity and help ensure that historically under-represented communities are enrolled in clinical studies. It requires drug sponsors to demonstrate that a diverse patient body was included in clinical trials for the drug. This may help to mitigate any potential power imbalance between patients and drug developers, as the industry is now mandated to consult a broad group of individuals to both inform their study design and test their products. While this does not directly reduce the possibility for conflict of interest, it is clear that the FDA is taking much-needed steps toward accountability and transparency in the clinical trial design and implementation process, and clinical trial sponsors will hopefully rise to meet these new standards.

Using well-established partnerships between patients and researchers, CAB members pinpointed strategies to create clinical trials that are inclusive, diverse, and patient-centered. Suggestions included creating easy-to-understand communication materials, encouraging communication between trial participants and researchers, and providing a human face to the research. CAB members felt that people’s humanity can be lost when taking part in a study, as researchers can sometimes treat participants according to their perceived value to the study and their status as someone living with a particular health condition, rather than as whole and complex individuals (31). Collaboration between researchers and CABs can refine research outcomes by humanizing the work, uplifting patients’ voices and incorporating their preferences into research outcomes (31).

The outlined strategies suggested by participants have shown positive impacts across health industries and lead to improved interactions between researchers and clinical trial participants (26, 31–33). Overall, previous research has found that when researchers engaged with advocacy organizations, their research process became more collaborative, which better incorporated the priorities of those living with the diseases of focus (34).

## 5 Limitations

This study has some limitations. Due to the global nature of the CAB, cultural bias may have influenced the understanding and interpretation of the focus group questions, and language barriers may have prevented focus group participants from fully expressing their thoughts. An offer of co-authorship on this manuscript was extended to all focus group participants to ensure that their views were accurately reflected.

The only geographical regions not represented in this study are South and Central America, as there are no participants from these regions in the CAB, despite persistent efforts to recruit from them. Of existing members, all 23 were offered the opportunity to participate in one of the three focus group sessions, but due to the voluntary nature of participation, seven members chose not to participate.

Social desirability bias may have impacted participants’ responses. Researchers tried to mitigate this by having an external moderator lead the focus group sessions.

## 6 Conclusion

This CAB serves as a body of representatives for PLHB and D, with the goal of raising awareness about the challenges these groups face across the globe. This study captured the reflections of the inaugural cohort, which has been a new endeavor with efforts concentrated on establishing the CAB body’s structure and building its members’ knowledge and capacity. Researchers hope to repeat this process with future cohorts, the experiences of which may differ. There are lessons learned in terms of providing members the platform to effectively contribute to the design of diverse, inclusive, and patient-centered clinical trials, the development of high-quality treatments, and the possible advancement of a hepatitis B cure and more effective hepatitis delta therapeutics.

Participants felt that CAB participation has a positive impact on their lives, and they identified effective ways to support drug development, employing their unique position as people with lived experience to enhance communication between the drug development industry and the larger hepatitis B and D community. Because CAB members are trusted by their broader communities, they can serve as ambassadors, bridging the gap between scientists and PLHB and D. This could apply to both the communication of complex scientific information, and efforts to assuage some of the fears that may exist around clinical trial participation, thus reducing barriers to participation, encouraging enrollment and facilitating the development of safer and more effective treatments. Involving patients throughout the drug development process may also help build trust and acceptability of future treatments.

## Data Availability

The original contributions presented in the study are included in the article/Supplementary material, further inquiries can be directed to the corresponding author.
