# Supplementary material for: Global hepatitis B and D community advisory board: expectations, challenges, and lessons learned
Source: Front Public Health. 2024 Aug 26;12:1437502. doi: 10.3389/fpubh.2024.1437502 (PMC11381374; doi:10.3389/fpubh.2024.1437502)
Supplement: Supplementary file 1 [file Data_Sheet_1.docx]

Supplementary Material

# Supplementary Tables

Supplementary Table 1. Thematic table with participants’ responses, 2023.

This table includes additional participants’ quotes representing the thematic topics presented in the study.

| **Theme** | *Sub-theme* | *Quote* |
| --- | --- | --- |
| **Motivations** | *Representing PLHB and D* | “When I was first diagnosed, I went through a really dark period where I just wanted to pulled in on myself, if that makes sense. But then, I wanted to turn it around. I joined CAB because I wanted to be the voice of the people who hadn't moved on, who was still in that dark stage, who felt completely abandoned. And just to be a voice for the voiceless basically... I think I thought it was important to do something, turn it around and use my diagnosis as a force for good as opposed to just turning it on myself.” (U.K. participant)  “I would say my motivation is to really have a seat at the table in terms of Biopharma clinical trials, you know, they're spending a lot of resources investment to bring a cure and it's outside looking in, right? It's clinicians [and] scientists developing these protocols and things, but without like what matters or not fully including what matters to the patient experience and it's a diverse set of experience[s]... I see my role and my motivation to participate is to ensure that they are representative of patient, what patients care about and what matters.” (U.S. participant 4) |
|  | *Altruism* | “My motivation is just to spread the word and really to help people stop dying from Hep B which could be prevented. Also, to be honest, I'm really tired to be ongoing treatment for the rest of my life. And if I can help, oh, probably not for me. But for the next generation to have cure, why not? ... Okay, I cannot help myself. But if I can help the others, yeah, this is my motivation. I want to do something with my life. If I can make any change, it will be great, even small change.” (Bulgarian participant) |
|  | *Knowledge and Skill Building* | “I saw the advantage of joining international patient groups. I mean, the voice of I mean, in terms of policymaking, even international policies can make a change in our country. So, it's very important for me to get in touch locally and even outside the country or even regionally as well.” (Filippino participant)  “I really wanted to have a platform where I could share my personal experiences, where I could feel safe, and then get more knowledge [about] Hepatitis. And I saw CAB as an opportunity to really do that.” (Ghanaian participant) |
| **Experiences** | *Experience as a CAB Member* | “I was diagnosed with HBV three and half years ago and for a long, long time I felt I had no voice whatsoever. And I think being a member of CAB gives us that voice to speak out.” (U.K. participant) |
|  | *Global Team Membership* | “It just seems like it's a community. We all come together and united by one disease, unfortunately. But I think it's made us all strong and we look out for each other.” (U.S. participant 3) |
|  | *Building Knowledge* | “My goal here is just to basically learn more globally. So, from other people in other region[s] of the country or other countr[ies] that I can learn from them and understand their situation and be able to do my part as whatever I can contribute.” (U.S. participant 2)  “We mostly concentrate on trainings and discussion... and now we need to be engaged in our community and... how we can translate all what we said here on[to] the field.” (Cameroonian participant) |
| **Challenges** | *Personal Challenges* | “At times you find meetings are scheduled a bit late. And we are in different time zones.” (Ugandan participant)  “My challenge is to do things like the conference in Vienna would have been absolutely fabulous. I was invited and [it] would have been absolutely fabulous to have gone to it. But I couldn't because of the risk to me of air travel and meeting in a big conference center with a lot of people.” (U.K. participant) |
|  | *Industry Engagement Challenges* | “...if CAB has connection with those guys in the laboratories, maybe we can ask them what they need and maybe they need patient perspective and we can provide this, but without knowing what they need, how we can support them.” (Bulgarian participant) |
| **Suggestions** | *Increasing Interaction with Industry* | “If we're building relationships with Biopharma industry, we got to be thinking about relationships and not transactional events, engagements, meeting at a conference, COLDA or AASLD that's an event. But we really need to think about cultivating a relationship with their people and that might transcend a two-year term.” (U.S. participant 4) |
|  | *Unique CAB Contributions* | “We can help in the clinical trial by going through the trial itself and sharing what the outcomes or what happens during the trial and what are our expectations so they can develop a drug that would be more safe and efficacious.” (Filippino participant) |
|  | *Considerations for Industry* | “So I think for me, I want to talk to pharmaceutical companies developing those drugs that before they enter our country that patients should not be burdened to give their own medical results or be cut off from medication because after that, they throw the patient at us and then we have to find the patients their own support.” (Filippino participant) |

Supplementary Table 2. Codebook.

This table includes the codes used to analyze the qualitative data resulting from the three focus groups.

| **Existing Level of Understanding** | Participant discusses their understanding of the role of HBV and HDV CABs, including their understanding of the mission (their confidence in their understanding of the CAB mission) and CAB-related activities | |
| --- | --- | --- |
|  | **Perceived Importance of CAB** | Why participants believe the CAB is important |
|  | **Important CAB Roles** | What roles participants consider most important (such as stakeholder interaction) |
|  | | |
| **Motivations for joining the CAB** | Participant explains why they joined the CAB; discusses specific reasons or activities that piqued their interest, including: | |
|  | **Contributing to Finding a Cure** | Participant discusses their interest in being a part of finding a cure for HBV (or treatment for HDV) and having input in things like clinical trials and/or the drug development process |
|  | **Giving Voice to PLHB and D** | Participant discusses their desire to support people living with HBV and HDV, including advocacy & community education, (if participant explains how their lived experience motivated their wish to join the CAB to advocate for others) |
|  | | |
| **Challenges** | **Personal Challenges** | Participant discusses the challenges they personally faced as a member of the CAB |
|  | **Industry Engagement Challenges** | Participant discusses challenges they believe made it difficult to interact with the pharmaceutical industry (and other stakeholders) |
|  | | |
| **Disclosing HBV and HDV Status** | | Participant discusses their experience or reasoning why they worried / did not worry about disclosing their (their family members’) HBV/HDV status by joining the CAB |
|  | | |
| **Experience as a CAB member** | Participant discusses their overall experience, positive or negative, as a CAB member | |
|  | **Social and Emotional Support** | Participant discusses their feelings about social and/or emotional support as a CAB member |
|  | **Building Knowledge** | Participant discusses their perceptions of the provided educational resources and training materials (degree of ‘helpfulness’) |
|  | | |
| **Global Team Membership** | Participant discusses their experience of being a member of a global team (their experience as a new member) | |
|  | **Participating in a Global Team** | Participant discusses the factors that effectively helped (or hindered) their participation in the global team |
|  | | |
| **Suggestions** | Participant offers general CAB-related suggestions | |
|  | **Suggestions for Future CAB Members** | Participant offers advice to future CAB members (ask questions, speak up during meetings) |
|  | **Suggestions for Building CAB Capacity** | Participant discusses what they believe would empower them to effectively participate in CAB activities (i.e. suggestions for other supportive resources, training and participation certificates, assigning/establishing leadership roles/member responsibilities); future CAB activities (i.e. in-person meetings, advocacy opportunities, events calendars); expanding membership, succession (staggered terms, buddy system) |
|  | **Suggestions for Industry Engagement** | Participant discusses how the CAB should move forward (achieve its mission); CAB involvement in the drug development process (how to have a greater impact on drug development (HBV cure, HDV treatments)); how to elevate the patients’ role in CT design (patients’ act as “face of CT” to increase recruitment) |
|  | | |
| **Advice for Other Orgs** | | Participant provides ideas and advice to other patient organizations who are interested in establishing a CAB (including member recruitment, member/org interactions, avoiding errors) |
|  | | |
| **Interesting Themes** | | Participant discusses an idea/topic not related to any of the themes above, but can be insightful in future analysis (Government Advocacy; Hopes for the CAB (increased funding); Community Fear of CT Participation (low understanding of CT process/lack of knowledge)) |

# Supplementary Figures

Supplementary Figure 1. Focus Group Moderator Guide, 2023.

**Global Hepatitis B Community Advisory Board:**

**Expectations, Challenges, and Lessons Learned**

**Moderator guide**

Section I: Welcome Remarks (5 minutes)

Thank you for choosing to participate in our group discussion today. My name is [name], and I work with the Hepatitis B Foundation. We are here today to get your thoughts and opinions about being a member of the global hepatitis B and delta community advisory board (and we will call it CAB throughout the discussion). We are particularly interested in learning about your motivations for joining the CAB, challenges that you may have encountered in contributing to the CAB activities and mission, and lessons learned, especially in terms of working within a global team.

Before we begin, I’d like to let you know that this discussion will be audio recorded, so that we can accurately incorporate your feedback. Please remember to speak clearly and one at a time during the discussion.

Please note that the discussion today will be kept strictly confidential, and all the insights derived from the study will be represented in a collective format. Although we will not use your name or identity during the presentation of the results of this study, the known fact that you are a member of the hepatitis B and delta global CAB may lead to you being identified by research audiences.

I would also like to confirm that your participation is voluntary and that you are not obliged to answer any question you are not comfortable answering, and you are not obliged to finish this group discussion. Also please note that you don’t have to have your camera on, but if you can, it really helps facilitate the discussion.

Also please note that we have one of the Hepatitis B Foundation staff attending. [Name] will be taking notes, and keeping track of time, so we don’t take much of your time.

Finally, please remember during this discussion, there are no right or wrong answers. We want your honest thoughts and opinions. We only ask that we all be respectful of one another during the discussion.

Does anyone have any questions before we begin?

**We will first begin with an icebreaker question to get to know each other a little bit (5 minutes):**

– If you were to pick a superpower, what would it be?

**Section II: Let us first discuss the level of awareness about the role of the hepatitis B and Delta CAB**

1. Can you describe the mission/role of the CAB in your own words?
2. How confident/ comfortable are you describing the role/ mission of this CAB?
3. If you would name one role of the CAB that you think is the most important, what would it be (from your perspective)?

**Section III: Motivations to/ and expectations from joining the CAB**

1. What were your motivations to joining the CAB?

Answer prompts: (learn more about clinical trials, inform drug development and finding a cure for hepatitis B and delta, add the lived experience perspective to clinical research)

1. Before deciding to join the CAB, how did you feel about disclosing your hepatitis B or D status by being a member of a global hepatitis B or D advisory board?

Answer prompts: (I already had my status disclosed, the cause was empowering to have a role in finding a cure for hepatitis B and delta, etc.)

**Section IV: Learning/ participating, and challenges:**

1. What did you think about the training tools/ resources made available in the past year?

Answer prompts: (helpful to a degree, they had so much information that I don’t remember all of them, I learned a lot about clinical trials, I still need to learn about other topics related to clinical trials)

1. What other support do you think is needed to enable/ empower you to effectively participate (or fully engage) in CAB activities?

Answer prompts: (timings of the meetings, providing clear tasks to work on and achieve, developing leading roles that CAB members can take on and lead the CAB work in)

1. From your perspective, what are the challenges you personally face when participating in CAB activities?

Answer prompts: (my knowledge is less than the rest of CAB members, I was enthusiastic then I lost interest, I don’t feel connected with other CAB members, I am working within my own community and hardly find the time to effectively engage/ contribute)

1. What do you think is a challenge/ barrier for the CAB to interact with potential stakeholders?

Answer prompts: (funding some of our interactions with Pharma seems challenging, stakeholders don’t perceive our CAB as an effective/ powerful body or are unaware of the goals/activities of our CAB)

1. From your perspective, what do you think are important factors/ elements to help you effectively participate in a global team?

Answer prompts: (strong personal relations with other team members, feeling understood by other team members, feeling that other team members share similar perspectives about hepatitis B functional cure or hepatitis delta treatments)

1. What do you think are the unique characteristics (differences) that exist between the current CAB members? How do you think these variations help or hinder achieving the CAB mission?

Answer prompts: (language, culture, knowledge base, experience, access to care and treatment which may affect the sense of urgency to find a functional cure)

**Section V: Thoughts about drug development:**

1. What do you think the next steps would be for this CAB to achieve its mission?
2. How could the CAB help advance the development of a functional cure for hepatitis B and effective hepatitis delta treatments?

Answer prompts: (Interact more with industry, discuss treatment experiences & hopes amongst CAB members and summarize our shared experiences to present to industry, go to more conferences / presentations to learn more & establish connections with others)

1. How can the CAB effectively impact drug development?

Answer prompts: (bring our voices to drug development meetings, learn about other peoples’ experiences and advocate for those affected in meetings with industry)

1. What do you think could be patients’ role in clinical trials’ design and conduct?

**Section VI: Lessons learnt:**

1. If you are to advise other patient organizations (not necessarily hepatitis B) who are interested in establishing a CAB, what would be your advice regarding:
   1. Recruiting CAB members
   2. Interacting with CAB members
   3. Building the capacity and leadership of CAB members
   4. Maintaining knowledge base within the CAB (succession)
   5. One piece of advice about something to avoid/ a mistake/ error/ misconception
